# A nonlinear association of total cholesterol with all-cause and cause-specific mortality

**DOI:** 10.1186/s12986-021-00548-1

**Published:** 2021-03-10

**Authors:** Guo-dong He, Xiao-cong Liu, Lin Liu, Yu-ling Yu, Chao-lei Chen, Jia-yi Huang, Kenneth Lo, Yu-qing Huang, Ying-qing Feng

**Affiliations:** 1 Department of Cardiology, Guangdong Cardiovascular Institute, Guangdong Provincial People’s Hospital, Guangdong Academy of Medical Sciences, No. 106, Zhongshan Second Road, Yuexiu District, Guangzhou, 510080 China; 2 Department of Epidemiology, Centre for Global Cardio-Metabolic Health, Brown University, Providence, USA

**Keywords:** Total cholesterol, All-cause mortality, Cancer, Cardiovascular disease

## Abstract

**Background:**

The link between total cholesterol (TC) and all-cause and specific mortality has not been elucidated. Herein, we aimed to evaluate the effect of TC levels on all-cause, cardiovascular disease (CVD), and cancer mortality.

**Methods:**

All data analyzed were obtained from the National Health and Nutrition Examination Survey 1999–2014. The relationship between levels of TC and mortality was determined through Cox proportional hazard regression analysis coupled with multivariable adjustments. Two-piecewise linear regression models and Cox models with penalized splines were applied to explore nonlinear and irregular shape relationships. Kaplan–Meier survival curve and subgroup analyses were conducted.

**Results:**

The sample studied comprised 14,662 men and 16,025 women, categorized as 25,429 adults aged 18–65 and 5,258 adults over 65 years old. A total of 2,570 deaths were recorded. All-cause, cardiovascular, and cancer mortality showed U-curve associations after adjusting for confounding variables in the restricted cubic spline analysis. Hazard ratios (HRs) of all-cause and cancer mortality were particularly negatively related to TC levels in the lower range < 200 mg/dL, especially in the range < 120 mg/dL (HR 1.97; 95% CI 1.38, 2.83, HR 2.39; 95% CI 1.21, 4.71, respectively). However, the HRs of cardiovascular disease mortality in the range < 120 mg/dL were the lowest (HR 0.60; 95% CI 0.15, 2.42). In the upper range, a TC range of ≥ 280 mg/dL was correlated with mortality as a result of CVD and cancer (HR 1.31; 95% CI 0.87, 1.97 and HR 1.22; 95% CI 0.82, 1.79). The lowest cumulative survival rate of all-cause mortality was recorded in the lowest TC-level group, while the lowest cumulative survival rate of CVD mortality was recorded in the highest TC-level group.

**Conclusions:**

A nonlinear association of TC level with all-cause, cancer, and CVD mortality in the American population was observed, suggesting that too low or too high serum total cholesterol levels might correlate with adverse outcomes.

**Supplementary Information:**

The online version contains supplementary material available at 10.1186/s12986-021-00548-1.

## Background

Cardiovascular disease (CVD) and cancer remain the main causes of mortality globally. Over 17 million people die of CVD every year, with the US accounting for almost 1 million deaths alone [1]. Moreover, the prevalence of cancer is on the rise globally [2].

Cholesterol is essential for several cellular processes and also participates in the pathogenesis of CVD and cancer [3–6]. Evidence from recent animal studies has linked impaired intracellular cholesterol metabolism to the development of many diseases [7].

Prospective studies have explored the relationship between cholesterol and all-cause, CVD, and cancer mortality [6, 8]. However, findings from such studies have been unreliable due to inconsistent results [9–12], and some are limited by small sample sizes [13–15]. Therefore, the link between cholesterol and all-cause, CVD, and cancer mortality remains obscure [16–19].

Recently, there has been an increasing interest in understanding the impact of lower or higher cholesterol intake on the occurrence of different chronic diseases [20]. Moreover, the optimal range of TC required for good health outcomes is still unknown. To answer these questions, we evaluated the effect of TC levels on all-cause, CVD, and cancer mortality in a large, nationwide US cohort.

## Methods

### Study population

This cohort study was based on prospective data from the National Health and Nutrition Examination Survey (NHANES), a cross-sectional survey program conducted in the United States by the National Center for Health Statistics (NCHS) that continually assesses the health status of civilians who are not institutionalized [21]. This study represents an analysis of data from the 1999–2014 NHANES cycles, collecting data from representative samples by either conducting interviews or laboratory tests. The Institutional Review Board of the National Center for Health Statistics, CDC, approved the protocol used by the NHANES. All participants provided informed consent.

**Fig. 1:**
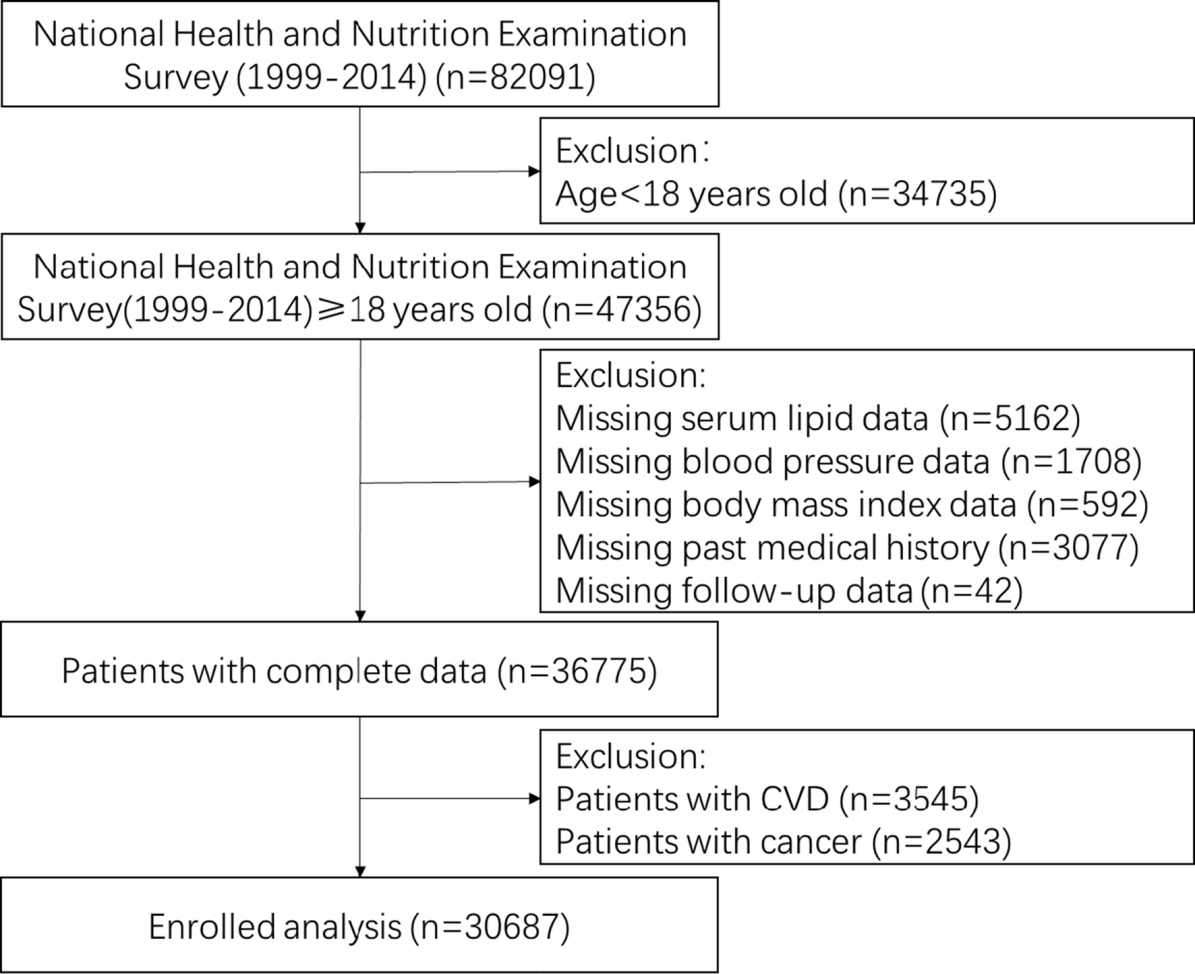
Study cohort

We extracted data based on the time when TC test data were obtained. The entire data integration process is presented in Fig. 1. In total, 82,091 participants were enrolled in the NHANES from 1999 to 2014. First, considering that participants < 18 years of age could introduce bias into the analysis, we excluded individuals under the age of 18 (N = 34,735). Second, we excluded individuals missing serum lipid data (N = 5162). In addition, individuals without blood pressure data (N = 1708), body mass index data (n = 592), past medical history (n = 3077), and follow-up data (n = 42) were all excluded. After missing entries were eliminated, 36,775 subjects with complete data remained. Then, we excluded individuals with CVD (n = 3545) and with cancer (n = 2543). Thus, 30,687 subjects, including 16,025 women and 14,662 men, were included in the final list.

### Baseline characteristics of data collection

Baseline characteristics, socio-demographic, and lifestyle factors, including age, gender, race, education level, marital status, smoking habits, body mass index (BMI), and energy intake, were provided by the participants during the household interviews [22]. Hypertension referred to an SBP (systolic blood pressure) of more than or equal to 140 mmHg or/and a DBP (diastolic blood pressure) of more than or equal to 90 mmHg. This was verified by the record of antihypertensive drug usage or history of hypertension reported by the patient. Diabetes was confirmed by fasting blood glucose ≥ 126 mg/dl, self-report, hemoglobin A1c ≥ 6.5%, or hypoglycemic drug usage.

Serum lipids were evaluated at the Lipoprotein Analytic Laboratory of Johns Hopkins University. Total cholesterol was measured by a colorimetric method, using a Hitachi 717 Analyzer (Boehringer Mannheim Diagnostics) from 1999 to 2005 and a Hitachi 912 (Roche Diagnostics) since 2006. Details regarding other laboratory tests can be obtained from the NHANES Laboratory. The quality control protocols and assurance met the 1988 Clinical Laboratory Improvement Act mandates.

### Mortality and follow-up

Mortality linkage methods used herein can be obtained from the NCHS. The anonymized data of individuals who participated in NHANES between 1999 and 2014 were allied to the longitudinal Medicare and mortality data based on the sequence number of NHANES. The NCHS classified mortality from CVD as follows: codes I00–I09 (acute rheumatic fever and chronic rheumatic heart diseases), code I11 (hypertensive heart disease), code I13 (hypertensive heart and renal disease), codes I20–I25 (ischemic heart diseases), codes I26–I51 (other heart diseases), codes I60–I69 (cerebrovascular disease), and codes C00-C99 (mortality from cancer), according to ICD-10.

### Data analysis

All the data analyses covered the complex, stratified, multistage, and cluster-sampling design (such as oversampling of some subpopulations) of NHANES are based on the strata, sample weights, and primary sampling units incorporated in the NHANES data.

TC levels were divided into six groups: < 120, 120–159, 160–199, 200–239, 240–279, and ≥ 280 mg/dL. The group which had the lowest all-cause mortality acted as a reference. A multivariable Cox proportional hazards model was applied to estimate the hazard ratios (HRs) and 95% confidence intervals (CIs). Cox proportional hazards regression models were utilized to understand the link between TC levels and total mortality and cause-specific mortality based on specific covariates: age, gender, and race in model II; plus education level, marital status, smoking habits, body mass index, systolic blood pressure, estimated glomerular filtration rate, high-density lipoprotein cholesterol, energy intake, comorbidities (hypertension and diabetes), and medication use (antihypertensive drugs, hypoglycemic agents, and lipid-lowering drugs) in model III. Continuous variables included: age, body mass index, blood pressure, estimated glomerular filtration rate, high-density lipoprotein cholesterol, and energy intake. Categorical variables consisted of gender, race, education level, marital status, smoking habits, comorbidities (hypertension and diabetes), and medication use (antihypertensive drugs, hypoglycemic agents, and lipid-lowering drugs).

To examine the irregular shape of the link between baseline TC and the risk of total mortality and cause-specific mortality, a Cox proportional hazards regression with a cubic spline functions model and smooth curve fitting (penalized spline method) were conducted. If nonlinear relationships were detected, two piecewise linear regression models were performed to elucidate how the associations differed by cut-off point. The cut-off points were estimated by trying all possible values and choosing the value with the highest likelihood.

The survival probability of the primary outcomes according to the different ranges of TC variability was calculated by using Kaplan–Meier curves for all-cause and cause-specific mortality.

To determine whether the relationship varied by age at the baseline (< 65 or ≥ 60 years), sex (female/male), race (white/non-white), or lipid-lowering drugs (yes or no), we performed subgroup analyses. Forest plots were drawn to visualize the different among subgroups.

For sensitivity analysis, we compared the difference between post-imputation data and pre-imputation data to understand the possible influence of our missing data approach on the results. Multiple imputation analyses were conducted to account for the missing data created from adjusting from multiple covariates. Five complete datasets were created (Additional file 1: Table S1). Analyses were performed in each of the five datasets and results were combined across imputations according to Rubin’s rules.

All analyses were administrated with R software 3.6.3 (R Core Team, Vienna, Austria), and the alpha level for significance in statistical analysis was set as 0.05 in this study.

## Results

### Baseline characteristics

The demographic features of the study participants based on the six TC levels are listed in Table 1. The average age of the 30,687 adults was 46.0 years, and about half of the sample were women (52.2%). The average level of cholesterol was 198.8 ± 42.2 mg/dL. Race, including Mexican American, other Hispanic, non-Hispanic White, non-Hispanic Black, and other races, accounted for 19.9%, 8.0%, 44.1%, 20.4%, and 7.6%, respectively. Almost three-quarters of participants self-identified as high school-educated or above. Half of the sample were married. 55.7% of the sample were not currently smokers. Women and individuals with higher education levels were more likely to have higher TC values. The levels of systolic and diastolic blood pressure increased across the TC levels; hypertension and diabetes accounted for approximately 37.6% and 13.5%, respectively. However, only 20.3% and 7.1% were treated with antihypertensive drugs and hypoglycemic agents.

**Table 1:** Demographic and clinical characteristics according to total cholesterol levels

Characteristic	Total	Total cholesterol, mg/dL						
		< 120	120–159	160–199	200–239	240–279	≥ 280	*p* Value
Number	30,687	385	4782	11,364	9486	3552	1118	
Age, years	46.0 ± 17.2	39.5 ± 18.6	41.0 ± 18.1	44.5 ± 17.1	48.7 ± 16.3	50.2 ± 16.0	49.6 ± 16.1	< 0.001
Gender, n (%)								< 0.001
Male	14,662 (47.8)	246 (63.9)	2417 (50.5)	5511 (48.5)	4469 (47.1)	1565 (44.1)	454 (40.6)	
Female	16,025 (52.2)	139 (36.1)	2365 (49.5)	5853 (51.5)	5017 (52.9)	1987 (55.9)	664 (59.4)	
Race, n (%)								< 0.001
Mexican American	6120 (19.9)	66 (17.1)	809 (16.9)	2273 (20.0)	2019 (21.3)	717 (20.2)	236 (21.1)	
Other Hispanic	2449 (8.0)	27 (7.0)	377 (7.9)	898 (7.9)	754 (7.9)	304 (8.6)	89 (8.0)	
Non-Hispanic White	13,536 (44.1)	136 (35.3)	1996 (41.7)	4910 (43.2)	4237 (44.7)	1710 (48.1)	547 (48.9)	
Non-Hispanic black	6252 (20.4)	126 (32.7)	1184 (24.8)	2398 (21.1)	1795 (18.9)	577 (16.2)	172 (15.4)	
Other race—including Multiracial	2330 (7.6)	30 (7.8)	416 (8.7)	885 (7.8)	681 (7.2)	244 (6.9)	74 (6.6)	
Education level, n (%)								< 0.001
Less than high school	8405 (27.4)	106 (27.6)	1212 (25.4)	3045 (26.8)	2669 (28.2)	1051 (29.6)	322 (28.9)	
High school or above	22,247 (72.6)	278 (72.4)	3563 (74.6)	8307 (73.2)	6809 (71.8)	2498 (70.4)	792 (71.1)	
Marital status, n (%)								< 0.001
Other	14,130 (46.6)	239 (62.7)	2545 (53.7)	5391 (48.0)	3981 (42.5)	1499 (42.9)	475 (43.6)	
Married	16,169 (53.4)	142 (37.3)	2192 (46.3)	5835 (52.0)	5386 (57.5)	1999 (57.1)	615 (56.4)	
Smoking, n (%)								< 0.001
No	17,083 (55.7)	201 (52.2)	2765 (57.9)	6403 (56.4)	5279 (55.7)	1855 (52.3)	580 (51.9)	
Yes	13,580 (44.3)	184 (47.8)	2014 (42.1)	4950 (43.6)	4199 (44.3)	1695 (47.7)	538 (48.1)	
Body mass index, kg/m^2^	28.6 ± 6.6	27.6 ± 7.0	28.0 ± 7.3	28.6 ± 6.7	28.9 ± 6.3	29.0 ± 5.9	28.8 ± 5.3	< 0.001
Systolic blood pressure, mmHg	122.9 ± 18.6	119.9 ± 17.9	118.9 ± 17.0	121.6 ± 17.8	124.6 ± 18.8	127.0 ± 20.2	128.0 ± 21.5	< 0.001
Diastolic blood pressure, mmHg	70.3 ± 13.0	65.8 ± 14.6	67.6 ± 12.2	69.9 ± 12.5	71.6 ± 12.9	72.2 ± 14.0	72.2 ± 14.7	< 0.001
eGFR, mg/min/1.73m^2^	91.3 ± 28.5	96.1 ± 31.6	93.5 ± 28.1	91.8 ± 27.1	89.9 ± 28.2	89.5 ± 30.8	94.0 ± 35.5	< 0.001
Energy intake, kcal	2170.5 ± 1029.4	2338.7 ± 1261.8	2217.8 ± 1065.4	2189.2 ± 1045.7	2141.4 ± 977.8	2131.6 ± 1044.2	2093.5 ± 980.6	< 0.001
Serum lipid level								
High density lipoprotein cholesterol								< 0.001
mg/dL	53.1 ± 15.9	41.8 ± 10.5	49.0 ± 12.3	52.3 ± 14.7	54.8 ± 16.6	56.3 ± 18.8	56.7 ± 20.4	
mmol/L	1.37 ± 0.41	1.08 ± 0.27	1.27 ± 0.32	1.35 ± 0.38	1.42 ± 0.43	1.46 ± 0.49	1.47 ± 0.53	
Total cholesterol								< 0.001
mg/dL	198.8 ± 42.2	108.4 ± 10.1	144.9 ± 10.4	180.4 ± 11.4	217.5 ± 11.3	255.6 ± 11.2	308.4 ± 40.3	
mmol/L	5.14 ± 1.09	2.80 ± 0.26	3.75 ± 0.27	4.67 ± 0.29	5.62 ± 0.29	6.61 ± 0.29	7.98 ± 1.04	
Comorbidities, n (%)								
Hypertension								< 0.001
No	19,146 (62.4)	251 (65.2)	3211 (67.1)	7357 (64.7)	5740 (60.5)	1967 (55.4)	620 (55.5)	
Yes	11,541 (37.6)	134 (34.8)	1571 (32.9)	4007 (35.3)	3746 (39.5)	1585 (44.6)	498 (44.5)	
Diabetes								< 0.001
No	26,552 (86.5)	302 (78.4)	3994 (83.5)	9914 (87.2)	8342 (87.9)	3074 (86.5)	926 (82.8)	
Yes	4135 (13.5)	83 (21.6)	788 (16.5)	1450 (12.8)	1144 (12.1)	478 (13.5)	192 (17.2)	
Treatment, n (%)								
Hypoglycemic agents								< 0.001
No	28,517 (92.9)	329 (85.5)	4233 (88.5)	10,598 (93.3)	8945 (94.3)	3379 (95.1)	1033 (92.4)	
Yes	2170 (7.1)	56 (14.5)	549 (11.5)	766 (6.7)	541 (5.7)	173 (4.9)	85 (7.6)	
Lipid-lowering drugs								< 0.001
No	27,777 (90.5)	328 (85.2)	4106 (85.9)	10,166 (89.5)	8766 (92.4)	3361 (94.6)	1050 (93.9)	
Yes	2910 (9.5)	57 (14.8)	676 (14.1)	1198 (10.5)	720 (7.6)	191 (5.4)	68 (6.1)	
Antihypertensive drugs								0.061
No	24,471 (79.7)	292 (75.8)	3771 (78.9)	9117 (80.2)	7561 (79.7)	2817 (79.3)	913 (81.7)	
Yes	6216 (20.3)	93 (24.2)	1011 (21.1)	2247 (19.8)	1925 (20.3)	735 (20.7)	205 (18.3)	
Outcomes, n (%)								
Cardiovascular disease mortality								< 0.001
No	30,200 (98.4)	383 (99.5)	4727 (98.8)	11,209 (98.6)	9316 (98.2)	3478 (97.9)	1087 (97.2)	
Yes	487 (1.6)	2 (0.5)	55 (1.2)	155 (1.4)	170 (1.8)	74 (2.1)	31 (2.8)	
Cancer mortality								0.124
No	30,123 (98.2)	376 (97.7)	4706 (98.4)	11,166 (98.3)	9305 (98.1)	3483 (98.1)	1087 (97.2)	
Yes	564 (1.8)	9 (2.3)	76 (1.6)	198 (1.7)	181 (1.9)	69 (1.9)	31 (2.8)	
All-cause mortality								< 0.001
No	28,117 (91.6)	349 (90.6)	4420 (92.4)	10,536 (92.7)	8626 (90.9)	3204 (90.2)	982 (87.8)	
Yes	2570 (8.4)	36 (9.4)	362 (7.6)	828 (7.3)	860 (9.1)	348 (9.8)	136 (12.2)	

eGFR, estimated glomerular filtration rate

Values are mean ± standardized differences or n (%)

During the NHANES follow-up period of up to 15 years from 1999 to 2014, 2,570 all-cause deaths were recorded, including 487 due to CVD and 564 cancer-related deaths.

### Associations between total cholesterol and mortality

Follow-up data on mortality can be obtained from the date the survey was started (median follow-up: 98.6 ± 54.0 months).

Table 2 shows the adjusted HRs (95% CIs) of specific and all-cause mortality in the six groups of TC levels. The associations of TC levels with incidence and mortality were non-linear. In models I and II, TC levels < 120 (mg/dL) were strongly linked to all-cause deaths, cardiovascular diseases, and cancer mortality. Similarly, TC levels ≥ 280 (mg/dL) were significantly related to all-cause deaths and deaths associated with cardiovascular diseases. An analysis with multivariable adjustments (model III) showed the HRs of total mortality and cancer mortality were still particularly negatively correlated with TC levels in the lower range of < 200 mg/dL, especially in the range of < 120 mg/dL (HR 1.97; 95% CI 1.38, 2.83 and HR 2.39; 95% CI 1.21, 4.71, respectively). However, the HRs of cardiovascular disease mortality in the range < 120 mg/dL were the lowest (HR 0.60; 95% CI 0.15, 2.42). In the upper range, a TC range of ≥ 280 mg/dL was correlated with mortality as a result of CVD (HR 1.31; 95% CI 0.87, 1.97). To ensure the robustness of our data analysis, we reanalyzed the association between total cholesterol and mortality using imputation data (Additional file 2: Table S2). Results of the sensitive analyses were consistent with above mentioned analysis.

**Table 2:** Multivariate Cox regression analysis of total cholesterol levels with cause-specific mortality

	Event/total	Event rate/ 1000 person-years	Model I	Model II	Model III
			HR (95%CI), P-value	HR (95%CI), P-value	HR (95%CI), P-value
All-cause mortality					
Total cholesterol (per 1 mmol/L increment)	2570/30,687	10.20	1.06 (1.03, 1.10) 0.0006	0.94 (0.90, 0.97) 0.0008	0.92 (0.89, 0.96) 0.0001
Total cholesterol group, mg/dL					
< 120	36/385	12.82	1.45 (1.04, 2.02) 0.0300	2.13 (1.53, 2.98) < 0.0001	1.97 (1.38, 2.83) 0.0002
120–159	362/4782	10.08	1.14 (1.01, 1.29) 0.0410	1.36 (1.20, 1.53) < 0.0001	1.35 (1.18, 1.53) < 0.0001
160–199	828/11,364	9.02	Reference	Reference	Reference
200–239	860/9486	10.70	1.17 (1.07, 1.29) 0.0010	0.94 (0.86, 1.04) 0.2232	0.93 (0.84, 1.03) 0.1558
240–279	348/3552	11.24	1.23 (1.08, 1.39) 0.0013	0.95 (0.84, 1.08) 0.4232	0.88 (0.77, 1.00) 0.0590
≥ 280	136/1118	13.29	1.43 (1.19, 1.72) 0.0001	1.16 (0.96, 1.39) 0.1188	1.07 (0.88, 1.29) 0.5114
P for trend			0.002	< 0.001	< 0.001
Cardiovascular mortality					
Total cholesterol (per 1 mmol/L increment)	487/30,687	1.93	1.18 (1.09, 1.27) < 0.0001	1.07 (0.98, 1.17) 0.1125	1.05 (0.96, 1.15) 0.2730
Total cholesterol group, mg/dL					
< 120	2/385	0.71	0.43 (0.11, 1.72) 0.2320	0.62 (0.15, 2.51) 0.5061	0.60 (0.15, 2.42) 0.4692
120–159	55/4782	1.53	0.92 (0.68, 1.25) 0.5933	1.07 (0.79, 1.46) 0.6622	1.01 (0.73, 1.40) 0.9466
160–199	155/11,364	1.69	Reference	Reference	Reference
200–239	170/9486	2.12	1.24 (1.00, 1.55) 0.0498	1.00 (0.81, 1.25) 0.9789	0.98 (0.78, 1.23) 0.8393
240–279	74/3552	2.39	1.40 (1.06, 1.85) 0.0175	1.11 (0.84, 1.46) 0.4804	1.02 (0.76, 1.37) 0.8938
≥ 280	31/1118	3.03	1.76 (1.19, 2.58) 0.0043	1.46 (0.99, 2.15) 0.0576	1.31 (0.87, 1.97) 0.2034
P for trend			< 0.001	0.208	0.410
Cancer mortality					
Total cholesterol (per 1 mmol/L increment)	564/30,687	2.24	1.03 (0.95, 1.11) 0.4600	0.93 (0.86, 1.01) 0.0764	0.93 (0.85, 1.01) 0.0909
Total cholesterol group, mg/dL					
< 120	9/385	3.21	1.51 (0.78, 2.95) 0.2243	2.07 (1.06, 4.03) 0.0335	2.39 (1.21, 4.71) 0.0121
120–159	76/4782	2.20	1.00 (0.77, 1.30) 0.9857	1.17 (0.90, 1.52) 0.2465	1.18 (0.89, 1.56) 0.2492
160–199	198/11,364	2.16	Reference	Reference	Reference
200–239	181/9486	2.25	1.03 (0.85, 1.27) 0.7409	0.87 (0.71, 1.06) 0.1743	0.85 (0.69, 1.05) 0.1294
240–279	69/3552	2.23	1.02 (0.77, 1.34) 0.8973	0.84 (0.64, 1.11) 0.2248	0.85 (0.64, 1.13) 0.2548
≥ 280	31/1118	3.03	1.37 (0.94, 2.00) 0.1042	1.19 (0.81, 1.74) 0.3660	1.22 (0.82, 1.79) 0.3276
P for trend			0.473	0.050	0.061

HR, hazard ratio; CI, confidence interval

Model I adjust for none

Model II adjust for age, gender, and race

Model III adjust for age, gender, race, education level, married, smoking, body mass index, systolic blood pressure, estimated glomerular filtration rate, high density lipoprotein cholesterol, energy intake, comorbidities (hypertension, and diabetes), and medication use (antihypertensive drugs, hypoglycemic agents, and lipid-lowering drugs)

**Fig. 2:**
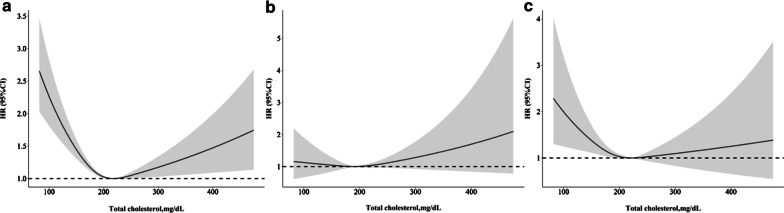
Spline analyses of all-cause (**a**), cardiovascular (**b**), and cancer (**c**) mortality by total cholesterol levels. (Adjusted for age, gender, race, education level, married, smoking, body mass index, systolic blood pressure, estimated glomerular filtration rate, high density lipoprotein cholesterol, energy intake, hypertension, diabetes, antihypertensive drugs, hypoglycemic agents, and lipid-lowering drugs)

Based on the restricted cubic spline analysis (Fig. 2), cardiovascular, cancer, and all-cause mortality showed U-curve associations after adjusting for confounding variables. These were largely similar to the TC categorical analysis. There were significant non-linear relationships between the levels of TC and specific and all-cause mortality.

We observed that the cutoff values were 172 mg/dL, 267 mg/dL, and 205 mg/dL for all-cause, cardiovascular disease, and cancer mortality, respectively, by using a two-piecewise linear regression model (Table 3). When TC levels were < 172 mg/dL and 205 mg/dL, a 1-unit decrease in the TC level was associated with a 40% and a 21% greater adjusted hazard ratio of all-cause and cancer mortality, respectively (HR 0.60; 95% CI 0.52, 0.70 and HR 0.79; 95% CI 0.67, 0.93, respectively). Cancer and all-cause mortality were not related when TC levels were ≥ 172 mg/dL and 205 mg/dL, respectively. When TC levels were ≥ 267 mg/dL, a 1-unit increase in the TC level was linked to a 38% greater adjusted hazard ratio of cardiovascular mortality (HR 1.38; 95% CI 1.09, 1.75).

**Table 3:** The results of two-piecewise linear regression model between total cholesterol and cause-specific mortality

	All-cause mortality	Cardiovascular mortality	Cancer mortality
	HR (95% CI) *p* value	HR (95% CI) *p* value	HR (95% CI) *p* value
Cutoff value, mg/dL	172(4.45 mmol/L)	267(6.90 mmol/L)	205(5.30 mmol/L)
< Cut-off value	0.60 (0.52, 0.70) < 0.0001	0.99 (0.89, 1.10) 0.8479	0.79 (0.67, 0.93) 0.0058
≥ Cut-off value	1.01 (0.96, 1.06) 0.8160	1.38 (1.09, 1.75) 0.0084	1.05 (0.92, 1.21) 0.4612
P for log likelihood ratio test	< 0.001	0.053	0.032

HR, hazard ratio; CI, confidence interval

The two-piecewise linear regression model were adjusted for age, gender, race, education level, married, smoking, body mass index, systolic blood pressure, estimated glomerular filtration rate, high density lipoprotein cholesterol, energy intake, comorbidities (hypertension, and diabetes), and medication use (antihypertensive drugs, hypoglycemic agents, and lipid-lowering drugs)

**Fig. 3:**
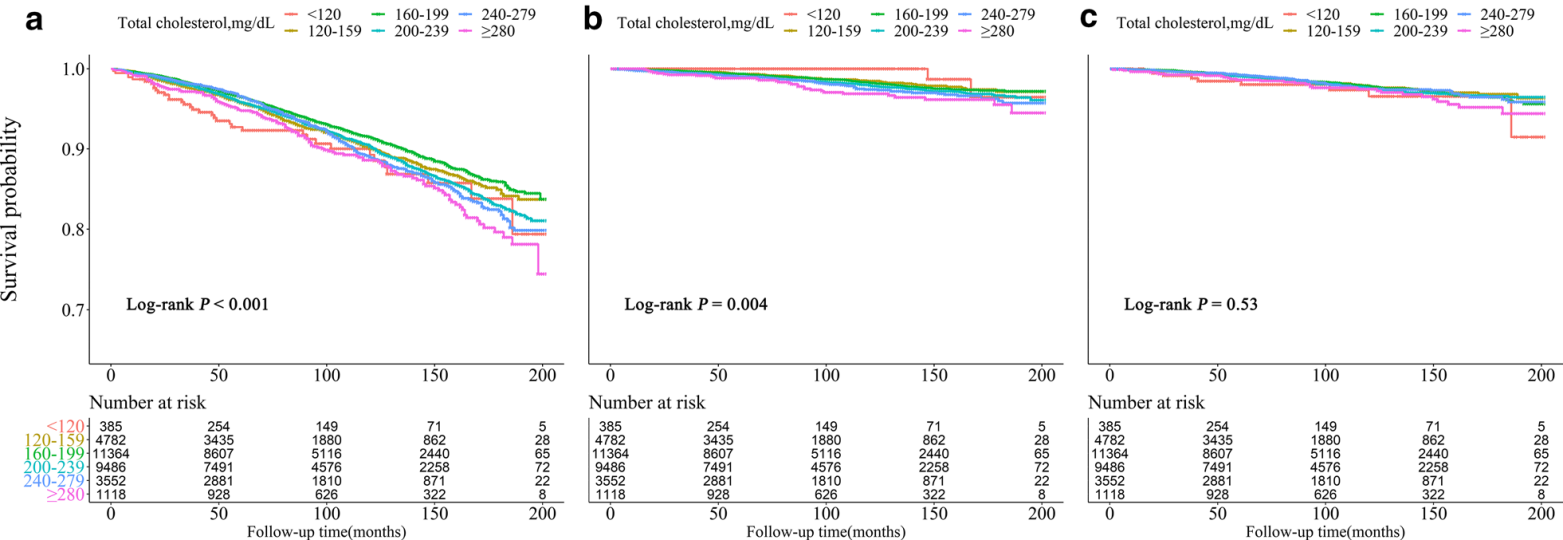
Kaplan–Meier survival curve for all-cause (**a**), cardiovascular (**b**), and cancer (**c**) mortality by total cholesterol

The Kaplan–Meier survival curve (Fig. 3) for all-cause, cardiovascular, and cancer mortality showed that the lowest cumulative survival rate of all-cause mortality was recorded in the lowest TC-level group, while the lowest cumulative survival rate of CVD mortality was recorded in the highest TC-level group.

### Subgroup analyses

**Fig. 4:**
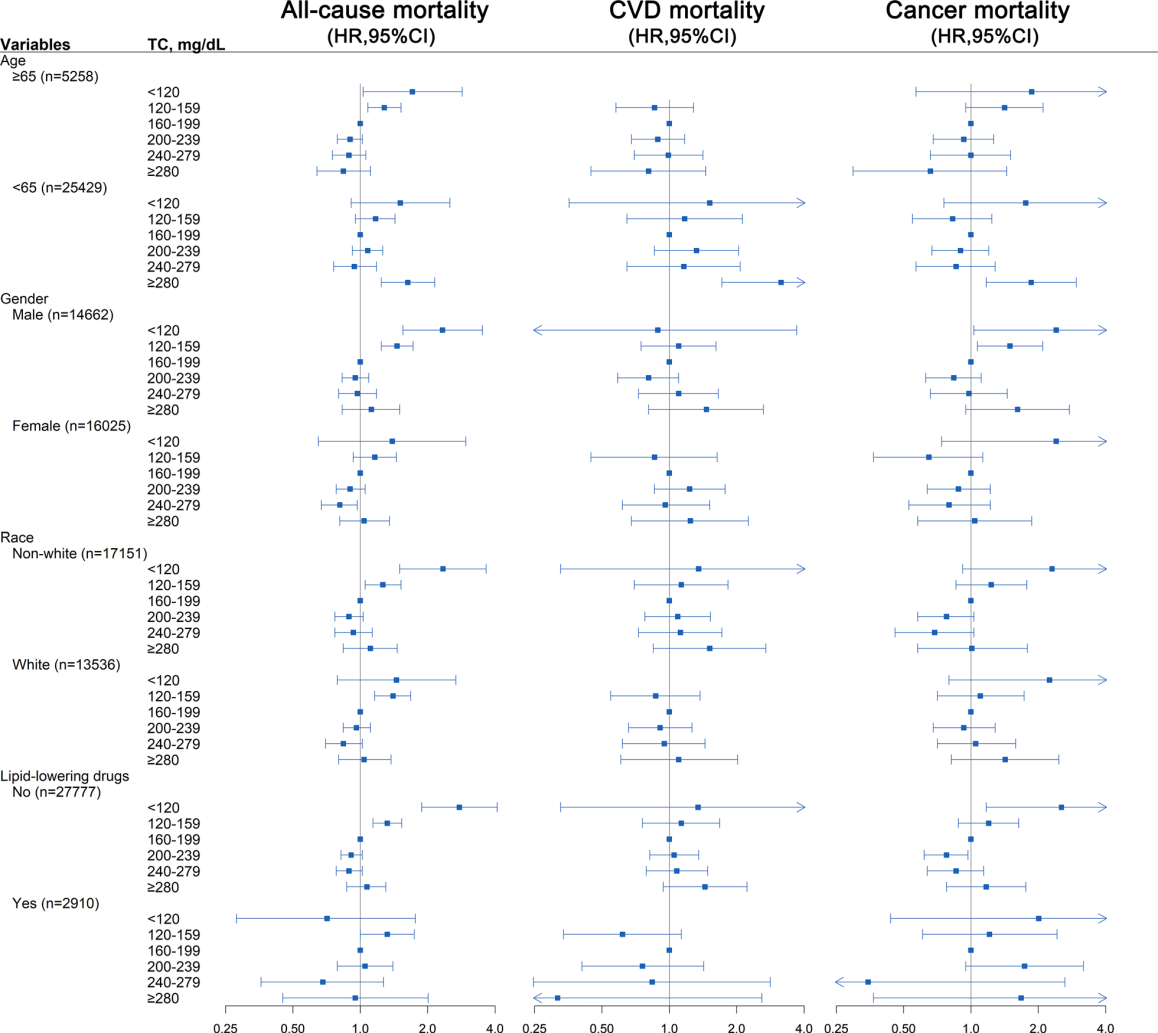
Forest plots of subgroups analyses. (age, gender, race, education level, married, smoking, body mass index, systolic blood pressure, estimated glomerular filtration rate, high density lipoprotein cholesterol, energy intake, hypertension, diabetes, antihypertensive drugs, hypoglycemic agents, and lipid-lowering drugs were all adjusted except the variable itself)

Subgroup analyses according to age, gender, race, and the use of lipid-lowering drugs were presented in Fig. 4.

Significant associations between the lowest range of TC level and all-cause mortality were observed in all subgroups except for using lipid-lowering drugs. When the range of TC level was 120–159 mg/dL, strong links were also observed in all subgroups.

The significant associations between the highest range of TC and cardiovascular mortality were observed in patients aged < 65 years, male, non-white, and without using lipid-lowering drugs.

The associations of the lowest range of TC range with cancer mortality were significant in all subgroups except for using lipid-lowering drugs. Interestingly, female with a TC range 120–159 mg/dL was inclined to have lower cancer mortality. Besides, the significant associations of TC level range 200–239 mg/dL with cancer mortality were observed in patients using lipid-lowering drugs. When TC was ≥ 280 mg/dL, TC was independently associated with cancer death in subjects aged < 65 years.

## Discussion

Herein, we revealed that there was a nonlinear association of TC with all-cause and specific-cause mortality, using data from the US NHNES. U-curve relationships between TC and mortality were found. Negative correlations in the lower TC range were strong in all-cause and cancer mortality, whereas positive correlations in the upper TC range were only found in CVD mortality. The occurrence of a TC range of < 120 mg/dL was strongly predictive of heightened death risk from all causes, whereas a TC range of ≥ 280 mg/dL was not, except with regard to CVD mortality.

Until recently, it was believed that TC is a biomarker of cholesterol metabolism and a significant contributor to atherosclerotic CVD, especially regarding the risk of CVDs. However, findings from previous studies have been inconsistent. Varied association shapes for TC versus all-cause mortality were obtained, including positive linear, U-curve, reverse-L-curve (or reverse-J-curve), and negative associations [16, 23, 24].

Current guidelines on cholesterol levels are largely based on CVD risk, and they all suggest a TC range of < 200 mg/dL as ideal. Interestingly, our study results also showed that the HRs of CVD mortality in the range of < 120 mg/dL were the lowest (HR 0.60; 95% CI 0.15, 2.42); however, a TC range of < 200 mg/dL was strongly linked to a higher risk of death from all causes, as well as cancer. It is no surprise that a TC range of < 200 mg/dL does not usually indicate good health with regards to the presence of other diseases. A recent national guideline on nutrition also did not recommend a reduction in dietary cholesterol [25]. Additionally, previous studies have mainly focused on elderly individuals. Our study showed a stronger effect size associated with a TC range of < 159 mg/dL at ages of < 65 years in all-cause mortality and a TC range of < 120 mg/dL at ages of < 65 years in cancer mortality. Besides, interpreting the connection between CVD mortality and cholesterol has been an intractable clinical challenge. In this study, the TC range of ≥ 280 mg/dL was positively related to cardiovascular mortality in patients aged < 65 years. Because individuals with low cholesterol levels exhibited higher rates of all-cause and cancer mortality, we postulated that low TC might partially reflected frailty. For CVD mortality, individuals aged below 65 years with a TC range of ≥ 280 mg/dL were more likely to die compared to aged 65 years or older. However, the association between low TC and a higher occurrence rate of serious adverse cardiovascular events in men aged ≥ 70 years has also been reported [26].

TC levels were usually classified into three categories: desirable (less than 200), borderline high (between 200 and 239), and high level (≥ 240 mg/dL). However, these categories were suggested according to the interactions between ischemic heart disease (IHD) and TC. In this study, 267 mg/dL and 205 mg/dL TC levels were linked to the lowest cardiovascular and cancer mortality rates in the spline analyses, respectively. Our data indicated that the optimal overall survival ranges were higher relative to those for IHD. Previous studies have shown a higher optimal BMI range for overall survival relative to IHD mortality [27]. Cholesterol levels may reflect the general health status but not the health status specific to CVD [28].

The association of low cholesterol levels with higher mortality may be explained by reverse causality. However, a study that involved long-term follow-up with the Japanese-American population indicated that people with low cholesterol levels that last for more than 20 years were likely to have the worst all-cause mortality. The study suggested that reverse causality was not likely to be fully responsible for the higher mortality linked to low cholesterol [29].

Besides, a key finding of our study regarding cancer suggested that low cholesterol could be linked to high rates of cancer-associated deaths. Although a few studies of statins indicated that statin therapy increases cancer incidence [30–32], it was still challenging to elucidate the connection between low cholesterol and cancer disease mortality. Previous studies on liver cancer reported an increased level of cholesterol [33, 34]. However, in this study, a higher rate of cancer mortality in the group with the lowest cholesterol was also significant. This suggested that screening the low cholesterol levels of patients with cancers is needed.

In addition to being a membrane component, cholesterol is a precursor to bile acids, certain fat-soluble vitamins, sex hormones (estradiol and testosterone), and steroid hormones (cortisone and aldosterone). It is involved in both the biogenesis of ligands in producer cells and signal reception in target cells. Recent structural, biochemical, and cell-biological studies have converged at the surprising model that a specific pool of plasma membrane cholesterol, termed accessible cholesterol, functions as a second messenger that conveys the signal between Patched 1 and Smoothened [7].

The effects of malnutrition, a risk factor for non-ischemic heart disease and cancer, might be the reason for this result [20, 35]. Therefore, a spontaneous decline in cholesterol levels may be a marker for a worsening health condition.

A large-scale, long-term complete follow-up study evaluating the influence of TC on all-cause and specific mortality are clear strengths of this study, and the large sample size used can ensure statistical efficiency. The NHANES database represented the entire US population, and this study has shown that low TC is a significant risk factor, not only in patients but also in the general population. Another advantage of this study is that it estimated all-cause, CVD, and cancer mortality risk related to TC levels as low as < 120 mg/dL. However, this study had some limitations. First, we could not understand the reason behind the link between TC and mortality risk in a cross-sectionally designed study. Second, we might have underestimated the risk associated with high cholesterol. Also, we could not verify that low cholesterol levels induced by statin increase mortality. Third, this study was based on a sample of American participants; as such, its findings may not be generalizable to other populations. Although the U-curve associations can apply to other ethnic groups, the level of relative risk related to TC, as well as the optimal TC range with the lowest mortality, might differ among various ethnic groups. Finally, physical activity and alcohol drinking were also important risk factors for all-cause and cause-specific mortality. However, we failed to adjust for these variables due to the lack of related data.

## Conclusions

In summary, a U-curve association of TC level with CVD, cancer, and all-cause mortality risks was observed in the American population, suggesting that cholesterol levels that are too low or too high in the serum might increase the likelihood of adverse outcomes. Besides, high TC levels were linked to CVD mortality, and positive correlations in the upper TC range were enhanced in age groups < 65 years and reduced in advanced age groups. However, a low TC level was linked to additional adverse outcomes. Our findings indicated that TC levels might be a critical risk factor in the general population, and TC levels < 200 mg/dL might not be indicative of good health. However, there is a need to conduct further studies to verify our findings. This will aid in the appropriate management of disorders linked to low TC levels and hence improve survival rates.

## Supplementary Information

**Additional file 1: Table S1.** Distributions of variables with missing data comparing observed complete case data to results from 5 imputed datasets with variables imputed from multiple imputation.

**Additional file 2: Table S2.** The comparison of multivariate cox regression analysis between pre-imputation data and post-imputation data.

## Data Availability

Not applicable.

## Abbreviations

TC: Total cholesterol
CVD: Cardiovascular disease
NHANES: The National Health and Nutrition Examination Survey
NCHS: The national center for health statistics
SBP: Systolic blood pressures
DBP: Diastolic blood pressure
HRs: Hazard ratios
CIs: Confidence intervals
BMI: Body mass index
IHD: Ischemic heart disease

## References

[1] Benjamin EJ, Virani SS, Callaway CW, Chamberlain AM, Chang AR, Cheng S, Chiuve SE, Cushman M, Delling FN, Deo R (2018). Heart disease and stroke statistics-2018 update: a report from the american heart association. Circulation.

[2] Siegel RL, Miller KD, Jemal A (2020). Cancer statistics, 2020. CA Cancer J Clin.

[3] Lin X, Liu L, Fu Y, Gao J, He Y, Wu Y, Lian X: Dietary Cholesterol Intake and Risk of Lung Cancer: A Meta-Analysis. Nutrients. 2018;10. 10.3390/nu10020185 PMC5852761 29419756

[4] Berger S, Raman G, Vishwanathan R, Jacques PF, Johnson EJ (2015). Dietary cholesterol and cardiovascular disease: a systematic review and meta-analysis. Am J Clin Nutr.

[5] Soliman GA: Dietary Cholesterol and the Lack of Evidence in Cardiovascular Disease. Nutrients. 2018;10. 10.3390/nu10060780 PMC6024687 29914176

[6] Kuzu OF, Noory MA, Robertson GP (2016). The role of cholesterol in cancer. Cancer Res.

[7] Radhakrishnan A, Rohatgi R, Siebold C (2020). Cholesterol access in cellular membranes controls Hedgehog signaling. Nat Chem Biol.

[8] Gowdy KM, Fessler MB (2013). Emerging roles for cholesterol and lipoproteins in lung disease. Pulm Pharmacol Ther.

[9] Kubota Y, Iso H, Date C, Kikuchi S, Watanabe Y, Wada Y, Inaba Y, Tamakoshi A (2011). Dietary intakes of antioxidant vitamins and mortality from cardiovascular disease: the Japan collaborative cohort study (JACC) study. Stroke.

[10] Osganian SK, Stampfer MJ, Rimm E, Spiegelman D, Manson JE, Willett WC (2003). Dietary carotenoids and risk of coronary artery disease in women. Am J Clin Nutr.

[11] Agudo A, Cabrera L, Amiano P, Ardanaz E, Barricarte A, Berenguer T, Chirlaque MD, Dorronsoro M, Jakszyn P, Larrañaga N (2007). Fruit and vegetable intakes, dietary antioxidant nutrients, and total mortality in Spanish adults: findings from the Spanish cohort of the European prospective investigation into cancer and nutrition (EPIC-Spain). Am J Clin Nutr.

[12] Buijsse B, Feskens EJ, Kwape L, Kok FJ, Kromhout D (2008). Both alpha- and beta-carotene, but not tocopherols and vitamin C, are inversely related to 15-year cardiovascular mortality in Dutch elderly men. J Nutr.

[13] Genkinger JM, Platz EA, Hoffman SC, Comstock GW, Helzlsouer KJ (2004). Fruit, vegetable, and antioxidant intake and all-cause, cancer, and cardiovascular disease mortality in a community-dwelling population in Washington County Maryland. Am J Epidemiol.

[14] Weng LC, Yeh WT, Bai CH, Chen HJ, Chuang SY, Chang HY, Lin BF, Chen KJ, Pan WH (2008). Is ischemic stroke risk related to folate status or other nutrients correlated with folate intake?. Stroke.

[15] Marniemi J, Alanen E, Impivaara O, Seppänen R, Hakala P, Rajala T, Rönnemaa T (2005). Dietary and serum vitamins and minerals as predictors of myocardial infarction and stroke in elderly subjects. Nutr Metab Cardiovasc Dis.

[16] Stamler J, Daviglus ML, Garside DB, Dyer AR, Greenland P, Neaton JD (2000). Relationship of baseline serum cholesterol levels in 3 large cohorts of younger men to long-term coronary, cardiovascular, and all-cause mortality and to longevity. JAMA.

[17] Casiglia E, Spolaore P, Ginocchio G, Colangeli G, Di Menza G, Marchioro M, Mazza A, Ambrosio GB (1993). Predictors of mortality in very old subjects aged 80 years or over. Eur J Epidemiol.

[18] Petersen LK, Christensen K, Kragstrup J (2010). Lipid-lowering treatment to the end? A review of observational studies and RCTs on cholesterol and mortality in 80+-year olds. Age Ageing.

[19] Lin YC, Lin YC, Chen HH, Chen TW, Hsu CC, Peng CC, Wu MS (2016). Different effect of hypercholesterolemia on mortality in hemodialysis patients based on coronary artery disease or myocardial infarction. Lipids Health Dis.

[20] Zhang X, Edwards BJ (2019). Malnutrition in older adults with cancer. Curr Oncol Rep.

[21] Johnson CL, Dohrmann SM, Burt VL, Mohadjer LK (2014). National health and nutrition examination survey: sample design, 2011–2014. Vital Health Stat.

[22] Chen TC, Parker JD, Clark J, Shin HC, Rammon JR, Burt VL (2018). National health and nutrition examination survey: estimation procedures, 2011–2014. Vital Health Stat.

[23] Petursson H, Sigurdsson JA, Bengtsson C, Nilsen TI, Getz L (2012). Is the use of cholesterol in mortality risk algorithms in clinical guidelines valid? Ten years prospective data from the Norwegian HUNT 2 study. J Eval Clin Pract.

[24] Yi SW, Yi JJ, Ohrr H: Total cholesterol and all-cause mortality by sex and age: a prospective cohort study among 12.8 million adults**.** Sci Rep 2019, 9**:**1596. 10.1038/s41598-018-38461-y PMC6367420 30733566

[25] DeSalvo KB, Olson R, Casavale KO (2016). **Dietary Guidelines for Americans**. Jama.

[26] Gnanenthiran SR, Ng ACC, Cumming R, Brieger DB, Le Couteur D, Waite L, Handelsman D, Naganathan V, Kritharides L, Blyth F (2020). Low total cholesterol is associated with increased major adverse cardiovascular events in men aged ≥70 years not taking statins. Heart.

[27] Whitlock G, Lewington S, Sherliker P, Clarke R, Emberson J, Halsey J, Qizilbash N, Collins R, Peto R (2009). Body-mass index and cause-specific mortality in 900 000 adults: collaborative analyses of 57 prospective studies. Lancet.

[28] Ko DT, Alter DA, Guo H, Koh M, Lau G, Austin PC, Booth GL, Hogg W, Jackevicius CA, Lee DS (2016). High-density lipoprotein cholesterol and cause-specific mortality in individuals without previous cardiovascular conditions: the CANHEART study. J Am Coll Cardiol.

[29] Schatz IJ, Masaki K, Yano K, Chen R, Rodriguez BL, Curb JD (2001). Cholesterol and all-cause mortality in elderly people from the Honolulu Heart Program: a cohort study. Lancet.

[30] Shepherd J, Blauw GJ, Murphy MB, Bollen EL, Buckley BM, Cobbe SM, Ford I, Gaw A, Hyland M, Jukema JW (2002). Pravastatin in elderly individuals at risk of vascular disease (PROSPER): a randomised controlled trial. Lancet.

[31] Sacks FM, Pfeffer MA, Moye LA, Rouleau JL, Rutherford JD, Cole TG, Brown L, Warnica JW, Arnold JM, Wun CC (1996). The effect of pravastatin on coronary events after myocardial infarction in patients with average cholesterol levels. Cholesterol and Recurrent Events Trial investigators. N Engl J Med.

[32] Alsheikh-Ali AA, Maddukuri PV, Han H, Karas RH (2007). Effect of the magnitude of lipid lowering on risk of elevated liver enzymes, rhabdomyolysis, and cancer: insights from large randomized statin trials. J Am Coll Cardiol.

[33] Okamura T, Tanaka H, Miyamatsu N, Hayakawa T, Kadowaki T, Kita Y, Nakamura Y, Okayama A, Ueshima H (2007). The relationship between serum total cholesterol and all-cause or cause-specific mortality in a 17.3-year study of a Japanese cohort. Atherosclerosis.

[34] Iso H, Ikeda A, Inoue M, Sato S, Tsugane S (2009). Serum cholesterol levels in relation to the incidence of cancer: the JPHC study cohorts. Int J Cancer.

[35] Khan HA, Alhomida AS, Sobki SH (2013). Lipid profile of patients with acute myocardial infarction and its correlation with systemic inflammation. Biomark Insights.

