# Supplementary material for: A nonlinear association of total cholesterol with all-cause and cause-specific mortality
Source: Nutr Metab (Lond). 2021 Mar 10;18:25. doi: 10.1186/s12986-021-00548-1 (PMC7945313; doi:10.1186/s12986-021-00548-1)
Supplement: Supplementary file 1 — Additional file 1: Table S1. Distributions of variables with missing data comparing observed complete case data to results from 5 imputed datasets with variables imputed from multiple imputation. [file 12986_2021_548_MOESM1_ESM.docx]

**Table S1** Distributions of variables with missing data comparing observed complete case data to results from 5 imputed datasets with variables imputed from multiple imputation

|  | Number with missing data | Complete case | Multiple imputation1 | Multiple imputation2 | Multiple imputation3 | Multiple imputation4 | Multiple imputation5 |
| --- | --- | --- | --- | --- | --- | --- | --- |
| Education level- high school or above, n (%) | 35 (0.11) | 21114 (73.0) | 22268 (72.6) | 22269 (72.6) | 22269 (72.6) | 22271 (72.6) | 22270 (72.6) |
| Marital status-married, n (%) | 388 (1.26) | 15457 (53.4) | 16407 (53.5) | 16409 (53.5) | 16387 (53.4) | 16394 (53.4) | 16405 (53.5) |
| Smoking, n (%) | 24 (0.08) | 12827 (44.3) | 13591 (44.3) | 13590 (44.3) | 13595 (44.3) | 13594 (44.3) | 13590 (44.3) |
| eGFR, mg/min/1.73m2 | 88 (0.29) | 90.93 ± 27.75 | 91.30 ± 28.50 | 91.30 ± 28.49 | 91.31 ± 28.50 | 91.30 ± 28.49 | 91.31 ± 28.52 |
| Energy intake, kcal | 1243 (4.05) | 2171.56 ± 1029.46 | 2168.75 ± 1027.17 | 2168.10 ± 1028.10 | 2167.81 ± 1026.39 | 2167.17 ± 1030.16 | 2167.07 ± 1029.80 |

Abbreviations: eGFR, estimated glomerular filtration rate.

Values are mean ± standardized differences or n (%).
