# Supplementary material for: A nonlinear association of total cholesterol with all-cause and cause-specific mortality
Source: Nutr Metab (Lond). 2021 Mar 10;18:25. doi: 10.1186/s12986-021-00548-1 (PMC7945313; doi:10.1186/s12986-021-00548-1)
Supplement: Supplementary file 2 — Additional file 2: Table S2. The comparison of multivariate cox regression analysis between pre-imputation data and post-imputation data. [file 12986_2021_548_MOESM2_ESM.docx]

**Table S2 The comparison of multivariate cox regression analysis between** **pre-imputation data and post-imputation data**

|  | Complete case  HR (95%CI), P-value | Multiple imputation  HR (95%CI), P-value |
| --- | --- | --- |
| n | 28936 | 30687 |
| All-cause mortality |  |  |
| Total cholesterol (per mmol/L increment) | 0.92 (0.89, 0.96) 0.0001 | 0.93 (0.89, 0.97) 0.0003 |
| Total cholesterol group, mg/dL |  |  |
| <120 | 1.97 (1.38, 2.83) 0.0002 | 1.97 (1.40, 2.76) 0.0001 |
| 120-159 | 1.35 (1.18, 1.53) <0.0001 | 1.34 (1.19, 1.52) <0.0001 |
| 160-199 | Reference | Reference |
| 200-239 | 0.93 (0.84, 1.03) 0.1558 | 0.94 (0.86, 1.04) 0.2268 |
| 240-279 | 0.88 (0.77, 1.00) 0.0590 | 0.90 (0.79, 1.02) 0.0936 |
| ≥280 | 1.07 (0.88, 1.29) 0.5114 | 1.08 (0.89, 1.29) 0.4402 |
| Cardiovascular mortality |  |  |
| Total cholesterol (per mmol/L increment) | 1.05 (0.96, 1.15) 0.2730 | 1.07 (0.98, 1.16) 0.1556 |
| Total cholesterol group, mg/dL |  |  |
| <120 | 0.60 (0.15, 2.42) 0.4692 | 0.52 (0.13, 2.12) 0.3648 |
| 120-159 | 1.01 (0.73, 1.40) 0.9466 | 1.03 (0.75, 1.40) 0.8765 |
| 160-199 | Reference | Reference |
| 200-239 | 0.98 (0.78, 1.23) 0.8393 | 1.02 (0.81, 1.27) 0.8850 |
| 240-279 | 1.02 (0.76, 1.37) 0.8938 | 1.05 (0.78, 1.39) 0.7596 |
| ≥280 | 1.31 (0.87, 1.97) 0.2034 | 1.34 (0.91, 1.99) 0.1399 |
| Cancer mortality |  |  |
| Total cholesterol (per mmol/L increment) | 0.93 (0.85, 1.01) 0.0909 | 0.92 (0.85, 1.00) 0.0585 |
| Total cholesterol group, mg/dL |  |  |
| <120 | 2.39 (1.21, 4.71) 0.0121 | 2.08 (1.06, 4.09) 0.0343 |
| 120-159 | 1.18 (0.89, 1.56) 0.2492 | 1.20 (0.92, 1.57) 0.1812 |
| 160-199 | Reference | Reference |
| 200-239 | 0.85 (0.69, 1.05) 0.1294 | 0.87 (0.71, 1.06) 0.1710 |
| 240-279 | 0.85 (0.64, 1.13) 0.2548 | 0.82 (0.62, 1.09) 0.1691 |
| ≥280 | 1.22 (0.82, 1.79) 0.3276 | 1.15 (0.79, 1.69) 0.4626 |

All analyses were adjusted for age, gender, race, education level, married, smoking, body mass index, systolic blood pressure, estimated glomerular filtration rate, high density lipoprotein cholesterol, energy intake, comorbidities (hypertension, and diabetes), and medication use (antihypertensive drugs, hypoglycemic agents, and lipid-lowering drugs).
